# Triboelectric Yarns with Electrospun Functional Polymer Coatings for Highly Durable and Washable Smart Textile Applications

**DOI:** 10.1021/acsami.1c00983

**Published:** 2021-03-30

**Authors:** Tommaso Busolo, Piotr K. Szewczyk, Malavika Nair, Urszula Stachewicz, Sohini Kar-Narayan

**Affiliations:** † Department of Materials Science and Metallurgy, University of Cambridge, CB3 0FS Cambridge, United Kingdom; ‡ Faculty of Metals Engineering and Industrial Computer Science, AGH University of Science and Technology, 30-059 Kraków, Poland

**Keywords:** triboelectric devices, smart textiles, energy harvesting, electrospinning, triboelectric yarn

## Abstract

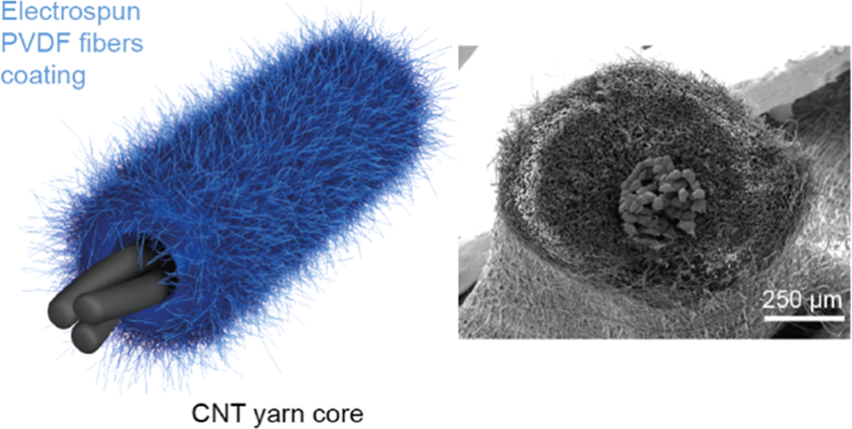

Triboelectric generators are excellent candidates for smart textiles applications due to their ability to convert mechanical energy into electrical energy. Such devices can be manufactured into yarns by coating a conductive core with a triboelectric material, but current triboelectric yarns lack the durability and washing resistance required for textile-based applications. In this work, we develop a unique triboelectric yarn comprising a conducting carbon nanotube (CNT) yarn electrode coated with poly(vinylidene fluoride) (PVDF) fibers deposited by a customized electrospinning process. We show that the electrospun PVDF fibers adhere extremely well to the CNT core, producing a uniform and stable triboelectric coating. The PVDF–CNT coaxial yarn exhibits remarkable triboelectric energy harvesting during fatigue testing with a 33% power output improvement and a peak power density of 20.7 μW cm^–2^ after 200 000 fatigue cycles. This is potentially due to an increase in the active surface area of the PVDF fiber coating upon repeated contact. Furthermore, our triboelectric yarn meets standard textile industry benchmarks for both abrasion and washing by retaining functionality over 1200 rubbing cycles and 10 washing cycles. We demonstrate the energy harvesting and motion sensing capabilities of our triboelectric yarn in prototype textile-based applications, thereby highlighting its applicability to smart textiles.

## 1 Introduction

Smart textiles and implantable electronics have enabled the development of a new generation of biosensors, diagnostic tools, and medical devices by bridging the gap between rigid electronics and soft human tissues.^1−7^ Continuous and noninvasive health monitoring, bioresorbable implants, and targeted drug delivery are just a few of the latest devices developed in this field.^8−10^ However, many of these technologies are limited by their power sources, which must be reliable, supply sufficient power, and withstand the harsh operating environment. Current batteries provide enough power but are rigid and require frequent charging or replacement, limiting their potential for wearable and implantable devices.^1,11^ A promising solution to address this issue is energy harvesting from the surrounding environment.^12^ This is a sustainable solution that can be adapted depending on the type of energy available, such as solar, thermal, or mechanical energy. Solar cells have been integrated into fabrics to harvest sunlight, thermoelectric devices have been used to exploit the thermal gradient between the body and the environment, and piezoelectric devices have been implemented to harvest energy from body movement.^12−17^ Mechanical energy harvesting is particularly promising owing to its abundance.^18^ It has been shown that ankle movement and heel strike can produce up to 67 and 20 W, respectively.^19^ Triboelectric generators offer a promising strategy to harvest mechanical energy due to their simple design, conformable structure, and high power output compared to piezoelectric devices.^20−23^ Triboelectric energy harvesting is based on the voltage generated by two surfaces periodically touching and separating due to the combined effect of contact electrification and electrostatic induction.^24,25^ Typically, triboelectric devices comprise one or more polymeric films attached to conducting electrodes, brought into periodic contact with each other. The choice of the triboelectric material is governed by its position on the triboelectric series,^26^ an empirical scale used to rank materials depending on their tendency to donate (tribopositive) or accept (tribonegative) charge during contact electrification.

Triboelectric devices have been conventionally fabricated on flexible polymer substrates and also recently integrated into textiles.^27−29^ Textile-based triboelectric generators have been developed using two strategies: (1) by attaching or printing the devices directly onto the textiles or (2) by fabricating functional yarns that are woven into the textile structure. The former method offers a simpler design and fabrication process but lacks the potential for seamless integration with textiles, which is an essential requirement to fulfill the wearable electronics potential. In contrast, triboelectric yarns offer a better opportunity for textile integration, despite having a more complex fabrication process. Although several yarn architectures have been demonstrated using metal electrodes and functional polymer coatings, the key challenges of the scalable fabrication process, durability, and integration with textile manufacturing processes remain unsolved.^12,30−32^

To address these challenges, it is necessary to design the yarn structure and fabrication process by considering both the energy harvesting and textile manufacturing requirements. The essential components of a triboelectric yarn are the conductive yarn (electrode) and the polymer coating (triboelectric material). The majority of triboelectric yarns that have been reported are based on metallic yarns or metalized fibers, which have good conductivity but limited durability and washing resistance.^28,33^ On the other hand, carbon nanotube (CNT) yarns offer exceptional specific strength and conductivity, allowing them to withstand aggressive textile manufacturing procedures.^34^ Furthermore, the CNT yarn strength enables facile interconnectivity between yarns and rigid components as they can be knotted to create both a mechanical and electrical connection, avoiding the need to use soldering, conductive epoxy, or other rigid joining methods.^35^ One of the key challenges for CNT yarns is biocompatibility, and several functionalization methods have been developed to address this problem.^36^ However, the functionalization process reduces the mechanical strength and electrical conductivity of the CNT yarn, limiting its potential for highly durable and washable triboelectric applications. Despite its many advantages, there have been no reports on triboelectric yarns based on CNT yarn electrodes thus far.

In this work, CNT yarns have been chosen as the conducting electrode onto which a suitable polymer coating is applied to fabricate a triboelectric generator. Polymer coating requirements include high triboelectric output, durability, and resistance to washing. The power output can be optimized by selecting materials at the far ends of the triboelectric series. Poly(vinylidene fluoride) (PVDF) is a highly tribonegative material that has been widely used in energy harvesting due to its ferroelectric and piezoelectric properties.^37,38^ In comparison to other fluorinated tribonegative polymers such as polytetrafluoroethylene, PVDF has higher mechanical strength and a simpler fabrication process. Furthermore, polymer crystallinity and surface potential can be tailored to enhance power output and adjust mechanical properties.^38,39^ An ideal one-step method to achieve such structural engineering is electrospinning. In this process, a high voltage is used to create an electric field between a nozzle and a collector. The polymer solution pushed through the nozzle is then drawn out into a polymer jet, which rapidly evaporates leading to a whipping motion that deposits polymer fibers onto the collector.^40^ Electrospinning is a scalable method for fiber production, which is widely used in industries such as filtration and tissue engineering. Recent work has demonstrated the potential of electrospun PVDF fibers as triboelectric materials both on a textile substrate and as a yarn.^41,42^

Here, we develop an extremely durable and washable triboelectric yarn based on a core–shell structure of CNT yarn and electrospun PVDF fibers. The materials, device structure, and fabrication process are optimized to achieve extensive fatigue, rubbing, and washing resistance while maintaining high power output. Our triboelectric yarn is able to withstand over 180 000 mechanical impact cycles and is able to not only maintain but also increase its power output after 200 000 fatigue cycles and 10 washes. In addition, the yarn demonstrates superior resistance to abrasion when tested using textile standards. Finally, we showcase proof-of-concept energy harvesting and sensing applications, highlighting the potential of our triboelectric yarn as a durable platform for smart textile and wearable technologies.

## 2 Results and Discussion

### 2.1 Design and Fabrication of the Triboelectric Yarn

**Figure 1:**
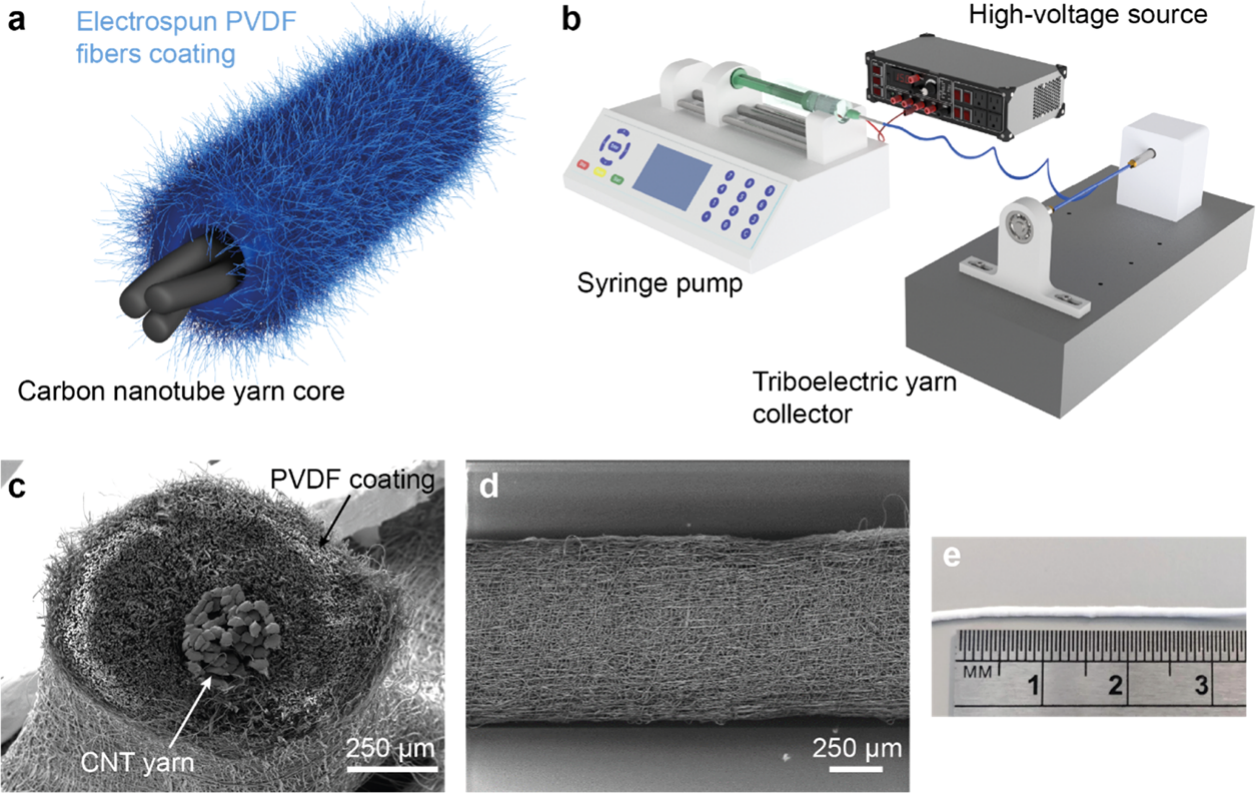
Fabrication and characterization of the triboelectric yarn. (a) Schematic of the triboelectric yarn core–shell structure. (b) Schematic of the fabrication setup for the triboelectric yarn. The setup is based on an electrospinning machine with a custom-made rotating collector. (c, d) SEM images across the cross section and along the length of the triboelectric yarn. (e) Photograph of the triboelectric yarn.

The proposed triboelectric yarn is based on a core–shell structure of a conductive CNT yarn coated with electrospun PVDF fibers (Figure 1a). The first step to fabricate the yarn was to create a bespoke electrospinning setup to accommodate the CNT yarn (Figure 1b). The syringe pump, nozzle, and the high-voltage power supply of the commercial electrospinning apparatus were not modified, but the standard drum collector was replaced by a CNT yarn clamped on either end with screw clamps. The CNT yarn was grounded and attached to a motor. During the electrospinning process, the nozzle was scanned along the rotating CNT yarn to create a uniform coating.

The scanning electron microscopy (SEM) images in Figure 1c,d show the cross section and the profile along the length of the triboelectric yarn. The triboelectric yarn had a diameter of approximately 850 μm, where the outer 575 μm consisted of PVDF electrospun fibers and the inner 275 μm were the CNT yarn. The image along the length of the yarn shows the fiber network on the surface of the yarn indicating a partial orientation in the fiber distribution along this direction (Figure 1d). The mean diameter of individual PVDF fibers was approximately 2.4 μm (Figures S1 and S2a). An image of the CNT yarn as received is shown in Figure S3. The photograph of the triboelectric yarn in Figure 1e shows the coating uniformity at the macroscale.

### 2.2 PVDF Coating Optimization

To achieve the desired combination of high durability and power output, we optimized the PVDF coating process. A crucial advantage of electrospinning is its ability to tailor the triboelectric yarn properties across several length scales. Macroscopically, the overall PVDF coating thickness and the electrospun fiber alignment can be controlled by modifying the deposition time and the collector rotation speed. The fiber diameter and surface morphology could be adjusted from microns to nanometers by varying the solvent type and polymer concentration.^43^ On the nanoscale, the voltage polarity and the relative humidity could be tuned to tailor the polymer crystallinity and the fiber surface chemistry.^44,45^

Given the large number of possible combinations of electrospinning parameters, in this work, we chose to focus on the effect of relative humidity on the triboelectric performance and fatigue resistance of the PVDF fibers.^46^ This parameter was selected because previous work showed its significant effect on polymer crystallinity and mechanical properties.^39^ Two types of PVDF fibers were produced, one using 60% relative humidity and another using 30% relative humidity, and referred to as PVDF60 and PVDF30, respectively. All other electrospinning parameters, including applied voltage and collector distance, were identical.

**Figure 2:**
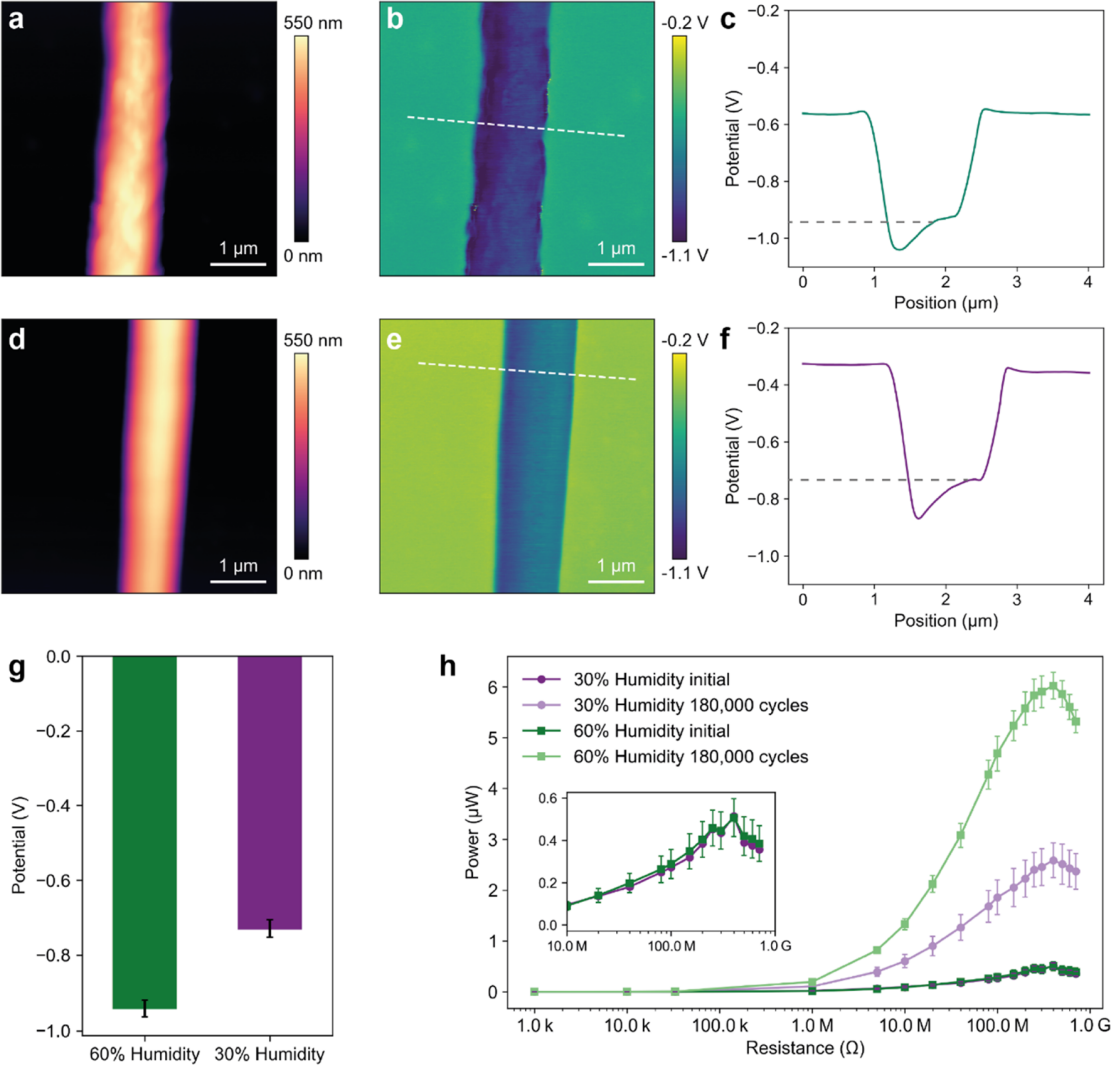
Tailoring coating fabrication process for durability and high triboelectric performance. (a) and (b) Surface topography and surface potential of PVDF fibers produced using 60% relative humidity during the electrospinning process. The dotted line is the location of the line scan. (c) Line scan of the surface potential of 60% relative humidity fibers. The dotted line indicates the surface potential of the fiber. (d) and (e) Surface topography and surface potential of PVDF fibers produced using 30% relative humidity during the electrospinning process. The dotted line is the location of the line scan. (f) Line scan of the surface potential of 30% relative humidity fibers. The dotted line indicates the surface potential of the fiber. (g) Surface potential of the fiber mats. Multiple measurements across several fibers were recorded and averaged. (h) RMS power output measured across several external resistances of PVDF mats produced with 30 and 60% relative humidities. The samples were measured as fabricated and after 180 000 cycles to evaluate fatigue performance. The inset shows the initial measurements.

The two samples were investigated using Kelvin probe force microscopy (KPFM), SEM, and mechanical energy harvesting characterization to study their properties across different dimensions. KPFM is a technique used to measure the surface potential and work function of a material and has been used to evaluate the position of the material on the triboelectric series.^47^ The results shown in Figure 2a–g indicated that PVDF60 and PVDF30 have average surface potentials of −0.94 ± 0.02 and −0.73 ± 0.02 V, respectively, as measured along the middle of each fiber. The slope in the surface potential seen on the line scan of both fibers was due to a measurement artifact; the real surface potential is indicated by a dotted line in Figure 2c,f. A lower surface potential means that the material has a higher work function and hence is more tribonegative.^47^ The KPFM data indicates that the PVDF60 fibers were more tribonegative than PVDF30 fibers.

The topography data in Figure 2a,d showed that PVDF60 fibers have a higher surface roughness than PVDF30 fibers. This is directly linked to the difference in humidity during the electrospinning process and has been previously reported.^46^ A high relative humidity during the electrospinning process of hydrophobic polymers (such as PVDF) is likely to cause porosity and surface roughness in fibers due to vapor-induced phase separation. During electrospinning, the water vapor interacts with the liquid polymer jet causing phase separation. This affects the fiber solidification process leading to the development of internal porosity and surface roughness. Recent work has shown that higher surface roughness in PVDF fibers can enhance the triboelectric power output.^48^

To investigate the effect of surface potential differences on the triboelectric power output, triboelectric generators were fabricated by depositing each fiber type on an aluminum foil. The devices were tapped against a Nylon 6 film (area 1.13 cm^2^), as shown in Figure S4. A gold electrode was sputter-coated on the noncontact side of the film. Initially, the power output data showed no significant difference between PVDF30 and PVDF60 devices, with both samples having a maximum root-mean-square (RMS) power of 0.5 μW at a 400 MΩ resistance (Figure 2h). Interestingly, after 180 000 tapping cycles, the PVDF60 device showed a power output almost 2.5 times higher than the PVDF30 device: the PVDF60 device produced a maximum RMS power output of 6 μW at 400 MΩ, whereas the PVDF30 device had a maximum RMS power output of 2.6 μW at 400 MΩ. In addition, the power output of both fiber types increased dramatically after 180 000 cycles by 12 and 5 times for PVDF60 and PVDF30, respectively.

The energy harvesting results were investigated by inspecting the sample surface using electron microscopy. PVDF60 fibers show higher fiber densities and no beads compared to PVDF30 fibers (Figure S5). PVDF60 fibers significantly deformed with large areas where individual fibers deformed into a film due to the mechanical impact. In contrast, PVDF30 fibers display minor deformation and minimal film formation; significant deformation is only seen on beads. This indicates that the PVDF60 fibers deform more easily than PVDF30 and thus increase their contact area after 180 000 cycles. The difference in mechanical properties between the fibers was demonstrated in previous work.^39,45^ PVDF60 fibers have 4.5 times higher elongation at failure and compliance than PVDF30 fibers because of their lower crystallinity and internal porosity.

The exact cause of the observed power output differences between PVDF60 and PVDF30 fibers after fatigue testing is related to a complex intersection of the aforementioned contact electrification and mechanical deformation phenomena that cannot be easily deconvoluted. However, it is clear that initially the differences in surface potential and mechanical properties between the PVDF60 and PVDF30 fibers did not strongly affect the triboelectric output of the devices. After 180 000 tapping cycles, the power output of both PVDF60 and PVDF30 fibers increased because of an increase in the contact area due to local aggregation of fibers into a film. It is likely that PVDF60 fibers showed a 2.5-fold increase in output power after 180 000 tapping cycles compared to PVDF30 fibers as larger areas of the sample turned into a film-like structure. PVDF60 fibers were used to fabricate the triboelectric yarn coating in subsequent experiments.

### 2.3 Energy Harvesting Characterization of Triboelectric Yarn

The energy harvesting performance of the CNT–PVDF core–shell triboelectric yarn was characterized using the device in vertical contact-separation mode with the setup shown in Figure S6. The tapping parameters (2 Hz and 10 N) were selected to simulate a heel strike.^19^ This is important as previous work either used unsuitable frequency for human motion (>4 Hz) or did not disclose the measurement conditions used.^49,50^ Additionally, we present all the energy harvesting data as RMS because it is representative of the equivalent steady DC output that the triboelectric generator can supply to a load. Importantly, the data shown is the average of three different samples that have been tested on different days (i.e., varying room temperature and relative humidity) to provide a more accurate representation of the performance of the triboelectric yarn across variable ambient conditions.

**Figure 3:**
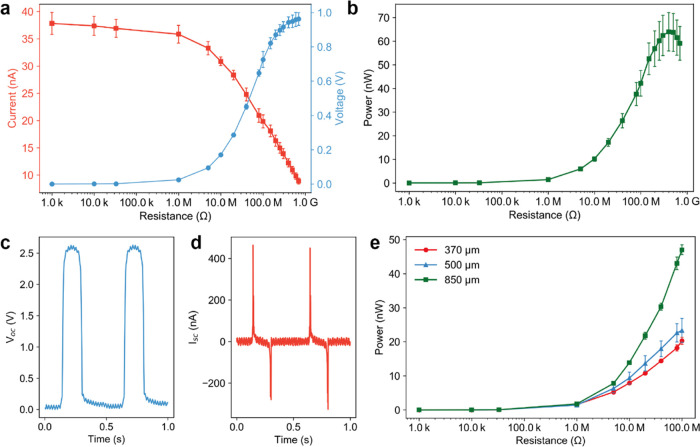
Electrical characterization of triboelectric yarn. (a) RMS voltage and current output of the triboelectric yarn across several resistors. (b) RMS power output of the triboelectric yarn across different resistors. (c) Open-circuit voltage of the triboelectric yarn. (d) Short-circuit current of the triboelectric yarn. (e) RMS power output of the triboelectric yarns with increasing coating thicknesses across several resistors.

Figure 3a,b shows the RMS voltage, current, and power output of the triboelectric yarn. The triboelectric yarn exhibited a maximum RMS power output of 72 nW at a 400M Ω resistance. The open-circuit voltage (*V*_oc_) and short-circuit current (*I*_sc_) were 2.6 V and 465 nA, respectively, as can be seen from Figure 3c,d. The working principle of the triboelectric yarn is shown in Figure S7. It is challenging to compare these results with other triboelectric yarns due to limited information about the mechanical motion used in the testing or contact area between materials, and the output power reported is often the peak power.^50,51^

The effect of coating thickness on the triboelectric power output of the yarn was investigated by fabricating samples with increasing thickness. The results shown in Figure 3e demonstrate that a thicker coating led to higher power output. This is related to the increase in contact area due to the larger coating thickness (Figure S8). However, the triboelectric yarn diameter should be balanced to ensure wearability and integration in textile manufacturing. Previously developed yarns had diameters over 5 mm, which is unsuitable for practical applications.^30,33^ Therefore, we selected a yarn diameter of approximately 850 μm. As a reference, the RMS power output of the CNT yarn (without the PVDF fiber coating) was 48 times lower than that of the triboelectric yarn (Figure S9).

### 2.4 Durability and Washing Resistance Characterization of the Triboelectric Yarn

**Figure 4:**
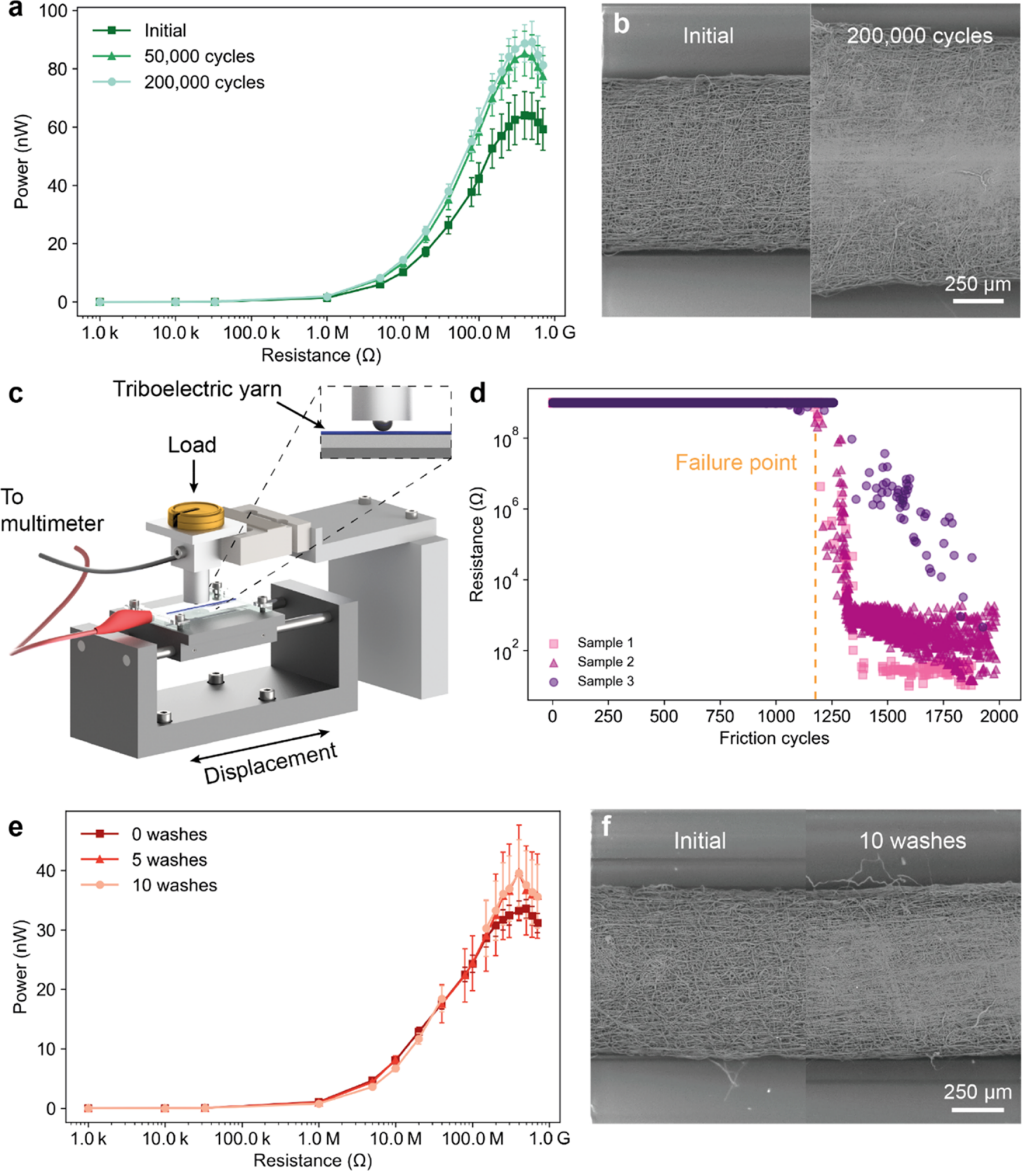
Durability and washing resistance characterization of the triboelectric yarn. (a) Fatigue evaluation of the triboelectric yarn. RMS power output of the triboelectric yarn across different resistors. (b) SEM images of the same triboelectric yarn as fabricated and after 200 000 tapping cycles. (c) Schematic of the friction testing setup. The resistance between the triboelectric yarn and the steel ball is continuously measured as the ball rubs the sample. The inset shows the contact between the triboelectric yarn and the steel ball. (d) Resistance between the triboelectric yarn and the steel ball across friction cycles. The dotted line highlights the failure point, where the coating begins to delaminate. (e) Washing resistance evaluation of the triboelectric yarn. RMS power output of the triboelectric yarn across different resistors. (f) SEM images of the same triboelectric yarn as fabricated and after 10 washes.

Durability and wear resistance are two key challenges that have yet to be addressed by current triboelectric yarns. Previously reported devices often limit their fatigue testing to a few thousand cycles under unrealistic testing parameters.^27,28,50,51^ Therefore, in this work, we put a strong emphasis on characterizing the fatigue and wear resistance using practical criteria and rigorous textile manufacturing standards. The results from fatigue tests shown in Figure 4a,b demonstrate the excellent performance of the triboelectric yarn. Its RMS power output not only did not degrade after 200 000 tapping cycles but instead was found to increase from an initial value of 72–92.5 nW after 50 000 cycles and 96 nW after 200 000 cycles. This increase can be attributed to the increase in the contact area due to deformation of PVDF fibers into a film-like structure. Upon inspection by SEM after fatigue testing, the coating did not present any signs of delamination or cracking (Figure 4b). The coating displayed wear marks where the contact occurred with the counter material, showing that the PVDF fibers coalesced into a film-like structure. In addition, the yarn diameter had been increased to 1.28 mm (67% increase) due to the deformation caused by repeated tapping. The combination of the PVDF fibers forming a film and the increase in yarn diameter caused an increase in the contact area between the triboelectric yarn and the Nylon 6 counter material, thus increasing the overall power output. The network structure created by the electrospun PVDF fibers is key to ensuring fatigue resistance as it provides high compliance, stress distribution, and toughness. A photograph of the yarn after 200 000 cycles is shown in Figure S10.

To the best of our knowledge, no previously reported triboelectric yarn has been able to retain and increase its power output after 200 000 fatigue cycles under realistic working conditions. Furthermore, we benchmarked the triboelectric yarn against other yarn-based triboelectric generators by measuring its peak power density (Figure S11). Our yarn exhibited a peak power density of 20.7 μW cm^–2^ after 200 000 cycles (for a contact area of 0.096 cm^2^), which is one of the highest reported in the literature.^52^

Measuring yarn wear resistance is challenging as there is no international standard available and no previous reports of triboelectric yarns explicitly investigated this property. Therefore, we developed a bespoke rubbing test to evaluate the wear resistance of the triboelectric yarn. The testing method is based on the color fastness to rubbing test (ISO 105-X12:2016), which is the textile industry benchmark for color resistance to abrasion.^53,54^ In this work, the abrasion testing was performed by mounting the yarn on a linear motor and repeatedly rubbing it against a steel ball attached to a static finger (Figure 4c). As the ball rubbed on the yarn, the electrical resistance between the CNT yarn and the ball was continuously monitored using a multimeter. Initially, there was an extremely high resistance between the two materials because they were separated by the insulating PVDF fibers. As the coating degraded due to rubbing, the resistance drop was used to quantify the wear behavior of the PVDF coating. The key parameters in this test were the applied stress and the number of rubbing cycles before failure. The ISO standard gives a pass when the fabric does not discolor after being rubbed at 0.05 MPa for 10 cycles. To demonstrate the wear resistance of our yarn, we increased the applied stress to 5 MPa and rubbed the yarn for over 2000 cycles.

Figure 4d shows the measured resistance between the CNT yarn and the ball across the rubbing cycles. Three triboelectric yarn samples were tested, and all started failing after approximately 1200 cycles as their resistance began to decrease. Figure S12 shows the triboelectric yarn surface after 2000 rubbing cycles, where it can be seen that the PVDF coating had been progressively removed, exposing the CNT yarn. The wear test highlighted the superior rubbing resistance of the triboelectric yarn, as it significantly exceeded the requirement of the ISO standard on both the applied stress and the number of rubbing cycles before failure.

To understand the reason behind the high wear resistance, we used Raman spectroscopy and Fourier-transform infrared spectroscopy (FTIR) to investigate the bonding between the CNT yarn and the PVDF fibers in the triboelectric yarn. Figure S13 shows the Raman spectra of the CNT yarn, electrospun PVDF fibers, and the CNT–PVDF core–shell triboelectric yarn. The Raman data did not show clear signs of chemical bonding between the CNT yarn and the PVDF fibers on the triboelectric yarn. In the case of chemical bonding, a shift in both the graphitic G and 2D peaks of the triboelectric yarn compared to those of the CNT yarn would be expected, but this was not observed (Figure S13b,c).^55^ The FTIR results showed that the triboelectric yarn peaks were almost exactly matched to the peaks from the PVDF (Figure S14). This further supported the evidence shown by the Raman spectra of little significant chemical bonding and instead points to the formation of a physical bond between the CNT yarn and the PVDF coating. Physical bonding is highly preferable because the chemical bonding of CNT and polymers often requires a functionalization process, which can alter the graphitic structure of CNT, thereby reducing its electrical conductivity and mechanical strength.^56^

CNT functionalization has also been used to address biocompatibility limitations. However, recent work has shown that nonfunctionalized CNT yarns fabricated using the same method as the triboelectric yarn appear to be biocompatible with a large range of cell types and in animal studies.^57^

For practical smart textile applications, it is essential to evaluate the washing resistance of the triboelectric yarn. The testing procedure was adapted from ISO 6330 7B and was comparable to previous studies.^51,58,59^ The setup comprised a beaker with a stir bar to simulate a washing machine, and no detergent was added. The triboelectric yarn was air-dried after every washing cycle. The triboelectric yarn power output was measured as fabricated and after 5 and 10 washing cycles. No significant change in the RMS power output was observed after washing (Figure 4e), suggesting that PVDF is not susceptible to degradation via hydrolysis and that the washing cycles did not have any significant effect on the triboelectric yarn stability and performance. The slight shift in the resistance at which maximum power is observed between 5 and 10 washing cycles may be due to small amounts of residual water within the yarn or minor yarn damage as a result of the washing cycle. The surface of the yarn showed typical wear caused by tapping but no particular signs of washing-related damage (see Figure 4f). The high washing resistance of the triboelectric yarn was expected considering that both PVDF and CNT are insoluble in water and highly mechanically stable, although we acknowledge that using alkaline detergent might cause minor degradation of the PVDF coating. The triboelectric yarn therefore showed excellent washing resistance, which is comparable to other high-performance triboelectric yarns.^51,60^

### 2.5 Smart Textile Energy Harvesting and Motion Sensing Applications

**Figure 5:**
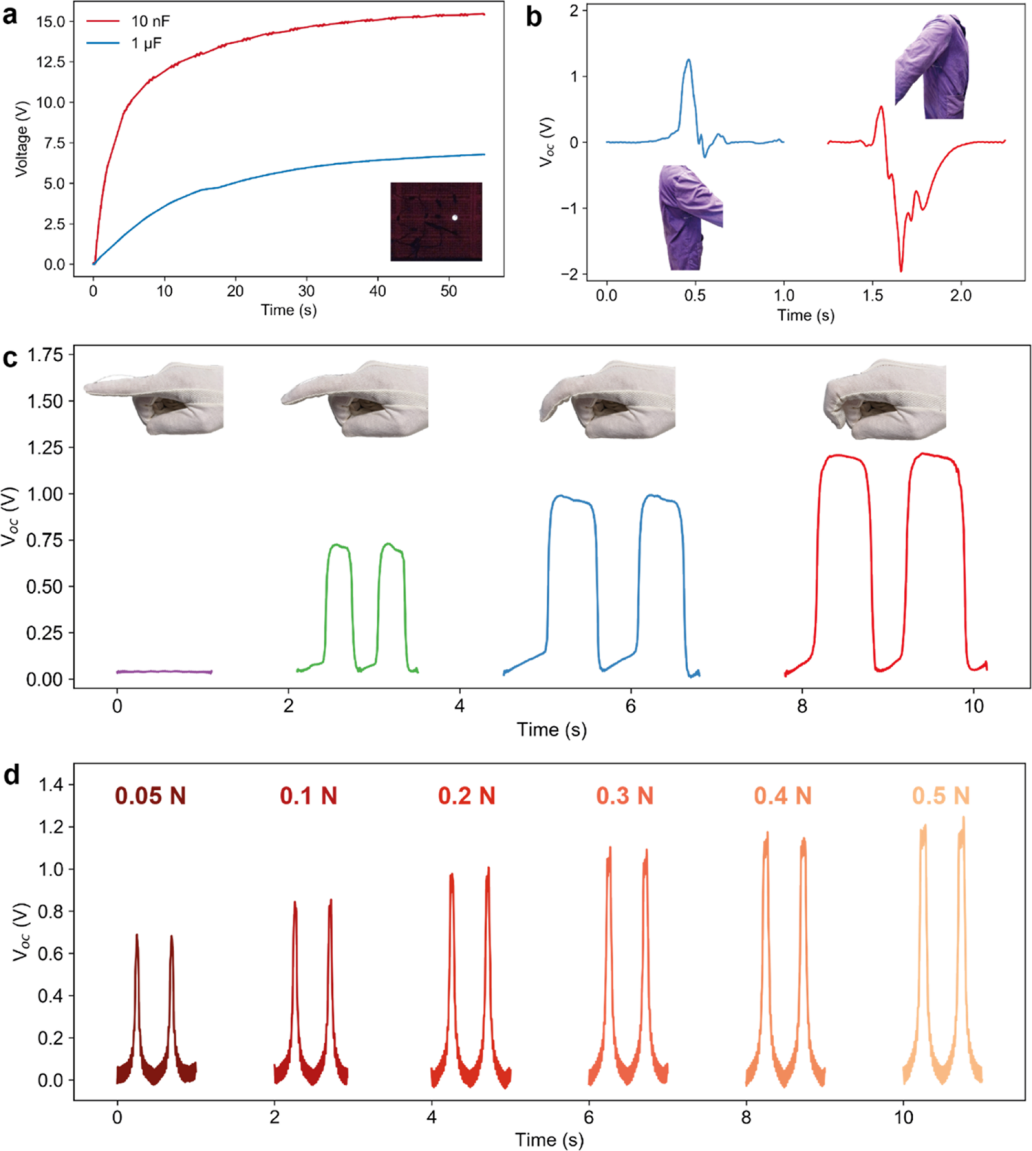
Energy harvesting and smart textile sensing application. (a) Capacitor charging curves using the triboelectric yarn of two different capacitors. A full-bridge rectifier was used to rectify the output. The inset shows the LED powered by the triboelectric yarn. (b) Arm motion sensing using the triboelectric yarn. The yarn was integrated into the lab coat by placing it under the armpit. The blue and red curves show, respectively, the *V*_oc_ caused by a forward and backward arm motion. The direction of the motion is shown in the insets. (c) Demonstrating the haptic potential of the triboelectric yarn integrated on a glove. The yarn (5 cm in length) was attached to the index finger of the glove. (d) Force sensing sensitivity of the triboelectric yarn. A load cell was used to measure the applied force.

After demonstrating the high durability of the triboelectric yarn, we integrated the device into textile substrates to demonstrate some potential applications in real-life situations. First, we showcase the energy harvesting potential by charging different capacitors (10 nF and 1 μF) and turning on an LED (Figure 5a). The triboelectric output was rectified using a bridge rectifier; the circuit is shown in Figure S15.

In Figure 5b,c, we demonstrate two applications of the triboelectric yarn as a wearable motion sensor. The sensor was used to determine the direction (forward and backward) of the arm movement by measuring *V*_oc_. The two voltage curves exhibited significantly different peak voltages and peak widths, making it easier for automatic software recognition. To showcase the potential of the triboelectric yarn as a haptic device, the yarn was attached to a glove and its output was recorded when bending the index finger, as can be seen in Figure 5c. The finger was repeatedly bent with three different angles to simulate different gestures. The amplitude of *V*_oc_ was found to increase gradually with the increasing bending angle, indicating the applicability of the yarn as a potential haptic sensor. It is important to note that the triboelectric yarn produced an easily measurable voltage despite being used in the single-electrode mode for the two applications (Figure S16). This mode is known to typically produce a significantly lower output voltage compared to vertical contact-separation mode. This further showcases the potential of the durable triboelectric yarn in different modes of operation and in different application scenarios ranging from wearable energy harvesting to sensing.

A great advantage of energy harvesting devices, and of triboelectric generators in particular, is that they can also be utilized as force sensors. The force sensing capability of the triboelectric yarn was characterized by measuring the minimum detectable force, as shown in Figure 5d. The yarn was repeatedly tapped using a linear motor with a load cell simultaneously measuring the tapping force. The minimum detectable force was 0.05 N with an associated *V*_oc_ of 0.6 V. The limiting factor for force detection was not the triboelectric yarn output but the load cell sensitivity range (max 0.05 N), suggesting that the triboelectric yarn could possibly have even higher force sensing capability. In addition, we then evaluated the effect of motion frequency on the triboelectric output of the yarn to understand its performance under different real-life scenarios (e.g., walking, running). The *I*_sc_ of the device showed a significant increase with tapping frequency (Figure S17).

## 3 Conclusions

In summary, we have developed a novel triboelectric yarn based on a CNT core and an electrospun PVDF coating that meets the durability and washability requirements of textile manufacturing. The CNT core overcomes the limitations of the traditional metal-coated yarn by providing an ideal combination of electrical conductivity, mechanical strength, and washing resistance. The electrospun PVDF fibers were engineered to produce a high output power and durable coating by tuning the relative humidity during the electrospinning process. The triboelectric yarn not only retained but also increased its energy harvesting performance by 33% after over 200 000 fatigue cycles with a peak power density of 20.7 μW cm^–2^. In addition, the yarn showed remarkable wear resistance by exceeding textile rubbing standards, withstanding over 1200 rubbing cycles. Washing tests did not damage the coating nor decrease the power output of the triboelectric yarn. Finally, we demonstrated real-life smart textile applications by showcasing both the energy harvesting and motion sensing potential of the triboelectric yarn. The durability, washing resistance, and scalability of the manufacturing process highlight the potential of the triboelectric yarn as a platform technology for smart textile applications.

## 4 Methods

### 4.1. Materials

The 24 wt % PVDF solution was prepared using PVDF (Mw = 275 000 g mol^–1^, Sigma-Aldrich) pellets dissolved in a 1:1 solution of dimethylacetamide (DMAc, analytical standard, Avantor) and acetone (analytical standard, Avantor). The solution was stirred for 4 h at a constant speed of 700 rpm on a hot plate set to 50 °C (IKA RCT basic). The CNT yarn was purchased from DexMat Inc. and used as received.

### 4.2. Electrospinning Process

Electrospinning was carried out using an IME Technologies EC-DIG electrospinner with a climate control system. The PVDF fiber mats were produced by applying +15 kV to the stainless needle located 180 mm from the grounded collector. The solution flow rate was set to 0.1mL min^–1^, and the time of electrospinning was 8 min. The electrospinning was carried out at a temperature of 25 °C and relative humidities of 30 and 60% to produce the two types of fibers PVDF30 and PVDF60.

The triboelectric yarn was fabricated using the same setup but with a modified collector to accommodate the CNT yarn. The electrospinning was performed by applying +15 kV to the stainless steel needle located 160 mm from the grounded CNT yarn. The flow rate of the solution for all samples was set to 0.06 mL min^–1^, and the CNT rotation speed was 500 rpm. The electrospinning temperature and relative humidity were 25 °C and 60%, respectively. The electrospinning time was tuned to obtain different coating thicknesses.

### 4.3. Microscopy

SEM was conducted using a Hitachi TM3000 with an accelerating voltage of 15 kV and a working distance of 3 mm. The triboelectric yarn cross-sectional investigation was performed using a Zeiss Merlin Gemini II. The sample was freeze-fractured in liquid nitrogen and imaged with an accelerating voltage of 3 kV and 150 pA current at a working distance of 3–8 mm. The freeze-fractured samples were gold-coated using a rotary-pump sputter coater (Q150RS, Quorum Technologies) with a 10 nm layer prior to imaging. The remaining samples were imaged uncoated. The fiber diameter measurements were performed utilizing ImageJ v1.5 software.

### 4.4. Scanning Probe Microscopy

The scanning probe measurements were performed using a Bruker MultiMode 8. The surface potential of PVDF fibers (diameter ≈ 0.5 μm) was measured via frequency-modulated KPFM. We used the conducting cantilever (MESP-RC-V2, Bruker) with a spring constant of 5 N/m, a resonance frequency of 150 kHz, and a cobalt-chromium (CoCr)-coated Si tip. The PVDF fibers were electrospun on a gold-coated silicon substrate for KPFM measurements. The KPFM measurements were carried out using sample bias. The data was inverted (to account for the bias being applied to the sample rather than the tip) for easier visualization.

### 4.5. Spectroscopy

Raman spectroscopy was carried out using a 785 nm laser and a Bruker Senterra Raman microscope. The scattered light was coupled into an ×20 long working distance objective, and a 1200 lines/mm grating was used. The laser power and acquisition time were varied between the CNT yarn, triboelectric yarn, and PVDF fiber mats to avoid sample damage. FTIR spectra were obtained using a Bruker Tensor 27 IR spectrometer equipped with an attenuated total internal reflection attachment.

### 4.6. Energy Harvesting Characterization

The energy harvesting performance of triboelectric devices was measured using a bespoke energy harvesting measurement setup. The devices were cyclically tapped against a Nylon 6 film using a linear motor (LinMot). The Nylon 6 film (Goodfellow) was a circular disc, 12 mm in diameter and 60 μm in thickness, and it was sputter-coated with gold on its noncontact side to create a vertical-separation triboelectric generator. The tapping parameters were 10 N and 2 Hz. The triboelectric yarn was supported using a poly(methyl methacrylate) holder. The output voltage and current were recorded via a multimeter (Keithley 2002) and a picoammeter (Keithley 6485), respectively. The measurements were recorded after 30 min of tapping to stabilize the electrical output. All energy harvesting results are an average of at least three samples measured across different days. The contact area was estimated by analyzing the SEM images using ImageJ software. The images were manually cropped to the region containing contacting fibers and then thresholded within the grayscale range such that white pixels represented the fibers and black pixels represented the background/empty space. Anomalous features that were not successfully eliminated by the automated background removal were manually deleted prior to area measurement. The contact area was determined from the area and area fraction of white to black pixels of each micrograph.

### 4.7. Wear Resistance Characterization

The triboelectric yarn wear resistance was measured using a custom-made setup. The testing conditions were adapted from the standard as the original test was performed on a fabric and not a single yarn. The yarn was fixed using clamps, and the entire assembly was attached to a linear motor. The rubbing was performed using a hardened chrome steel ball (5 mm diameter) attached to the static assembly. The yarn and the steel ball were connected to the multimeter (Keithley 2002) to measure the electrical resistance between them. The steel ball contact area was approximately 0.196 mm, and the applied force on the yarn was 0.98 N.

### 4.8. Washing Resistance Characterization

The washing resistance characterization was performed by placing the triboelectric yarn in a beaker full of water at 30 °C. The solution was stirred at 400 rpm using a magnetic stirrer. Each washing cycle comprised a 10 min washing period followed by air-drying.

## Supporting Information Available

SEM image of the PVDF fiber coating (Figure S1); fiber diameter histogram of the triboelectric yarn coating (Figure S2); SEM image of the CNT yarn (Figure S3); schematic of the triboelectric setup (Figure S4); SEM images of PVDF fibers (Figure S5); setup used to characterize the energy harvesting performance (Figure S6); working principles of the triboelectric yarn (Figure S7); SEM images of triboelectric yarns (Figure S8); RMS power output of the CNT yarn (Figure S9); photograph of the triboelectric yarn (Figure S10); peak power density (Figure S11); SEM image of the triboelectric yarn (Figure S12); Raman spectra of the CNT yarn, PVDF fibers, and the triboelectric yarn (Figure S13); FTIR spectra of the CNT yarn, PVDF fibers, and the triboelectric yarn (Figure S14); schematic of the rectifying circuit (Figure S15); schematic of the circuit used to detect arm motion and finger bending (Figure S16); and *I*_sc_ of the triboelectric yarn (Figure S17) (PDF)
