# Supplementary material for: Triboelectric Yarns with Electrospun Functional Polymer Coatings for Highly Durable and Washable Smart Textile Applications
Source: ACS Appl Mater Interfaces. 2021 Mar 30;13(14):16876–86. doi: 10.1021/acsami.1c00983 (PMC8045025; doi:10.1021/acsami.1c00983)
Supplement: Supplementary file 1 — am1c00983_si_001.pdf [file am1c00983_si_001.pdf]

Supporting information

Triboelectric yarn with electrospun functional polymer coatings for highly durable and washable smart textile applications

Tommaso Busolo[1], Piotr K. Szewczyk[2], Malavika Nair[1], Urszula Stachewicz[2], Sohini Kar-Narayan[1*]

[1] Department of Materials Science and Metallurgy, University of Cambridge, CB3 0FS, Cambridge, United Kingdom

[2] International Centre of Electron Microscopy for Materials Science and Faculty of Metals Engineering and Industrial Computer Science, AGH University of Science and Technology, Poland

* Corresponding author. Email: sk568@cam.ac.uk

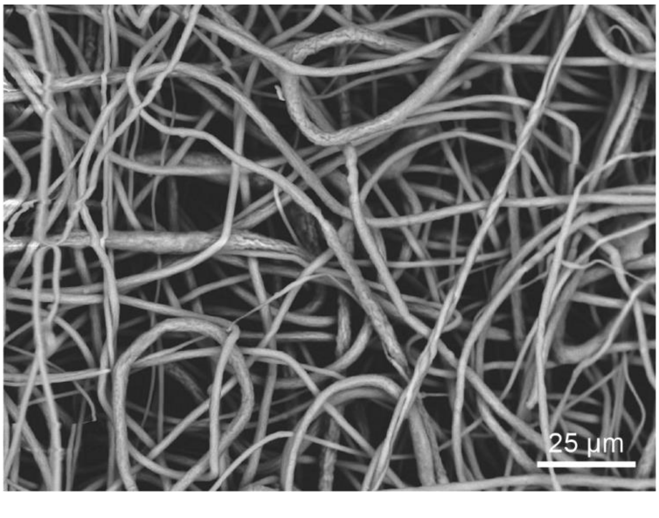

Figure S1. SEM image of the PVDF fibers coating of the triboelectric yarn.

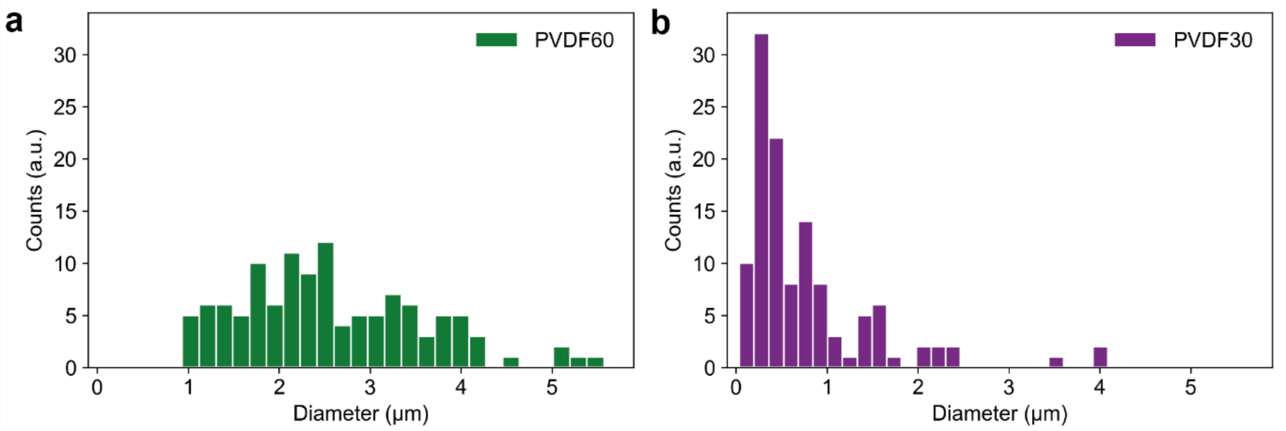

Figure S2. Fibers diameter histogram of triboelectric yarn coating. a) PVDF60. b) PVDF30

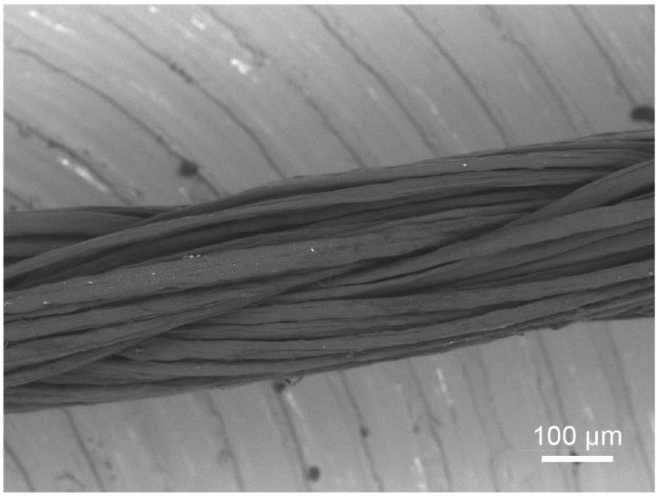

Figure S3. SEM image of the CNT yarn as received.

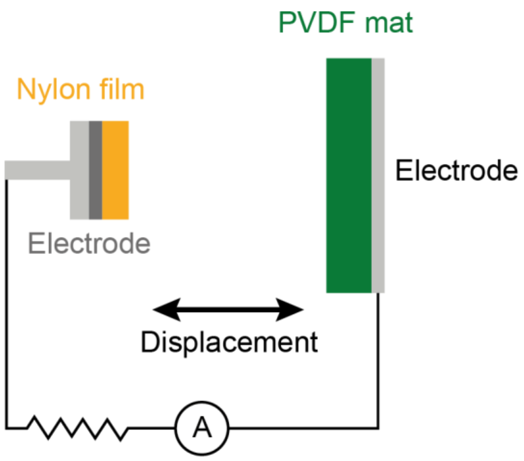

Figure S4. Schematic of the triboelectric setup used for electrical characterization of PVDF mats. The triboelectric devices were evaluated in vertical contact-separation mode.

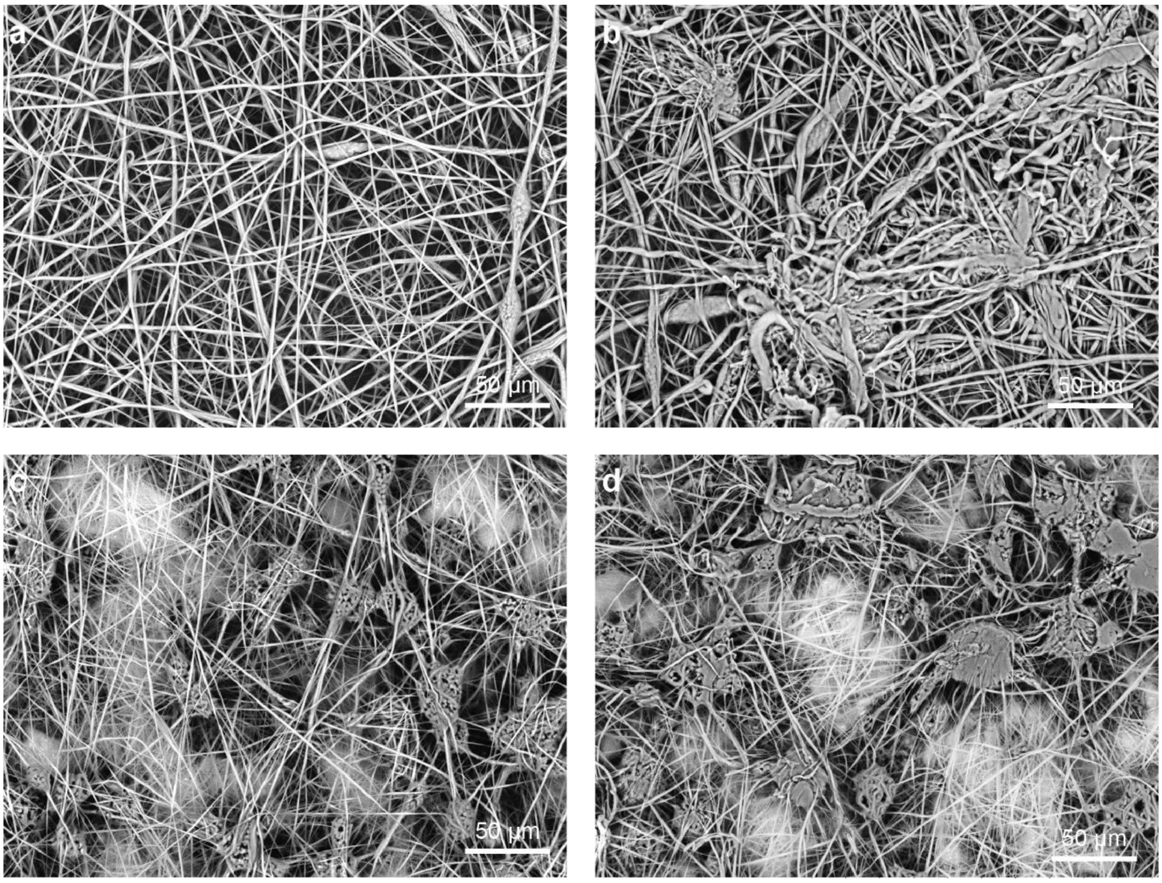

Figure S5. SEM images of PVDF fibers produced with 30% and 60% relative humidity before and after tapping. a) Image of PVDF60 fibers as fabricated. b) Image of PVDF60 fibers after 180,000 tapping cycles. c) Image of PVDF30 fibers as fabricated. d) Image of PVDF30 fibers after 180,000 tapping cycles.

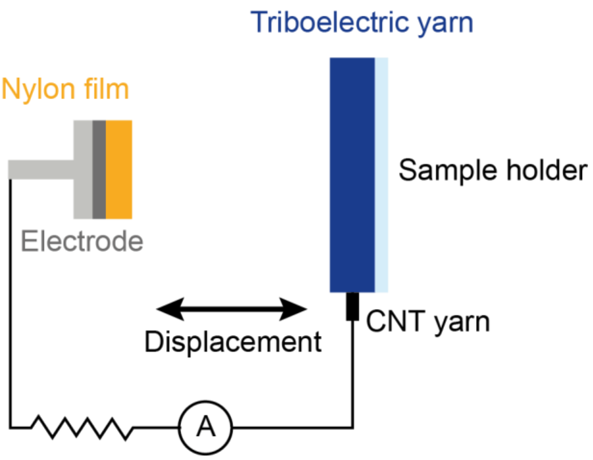

Figure S6. A schematic of the setup used to characterize the energy harvesting performance of the triboelectric yarn.

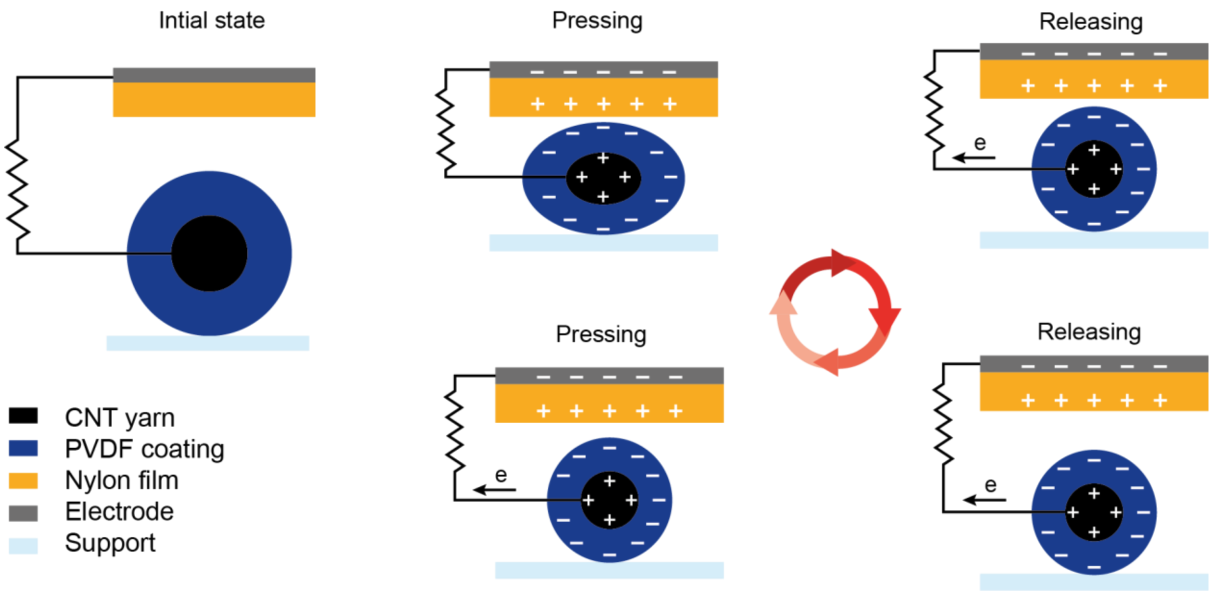

Figure S7. Working principles of the triboelectric yarn.

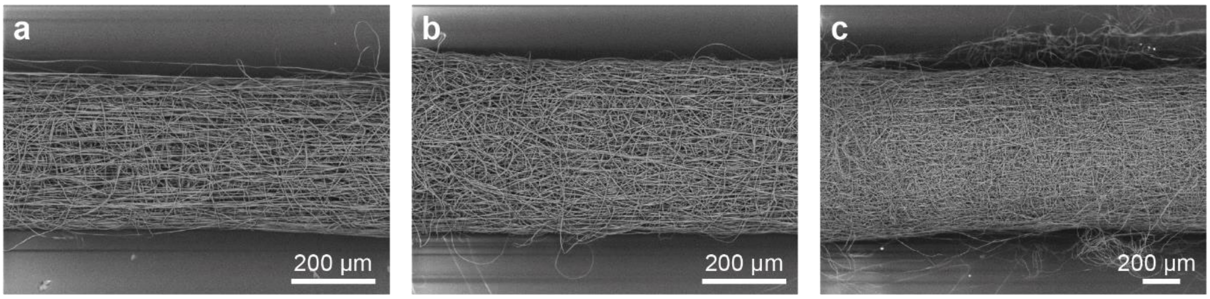

Figure S8. SEM images of triboelectric yarns with varying coating thickness. a) The yarn coating thickness is 370 μm. b) The yarn coating thickness is 500 μm. c) The yarn coating thickness is 850 μm.

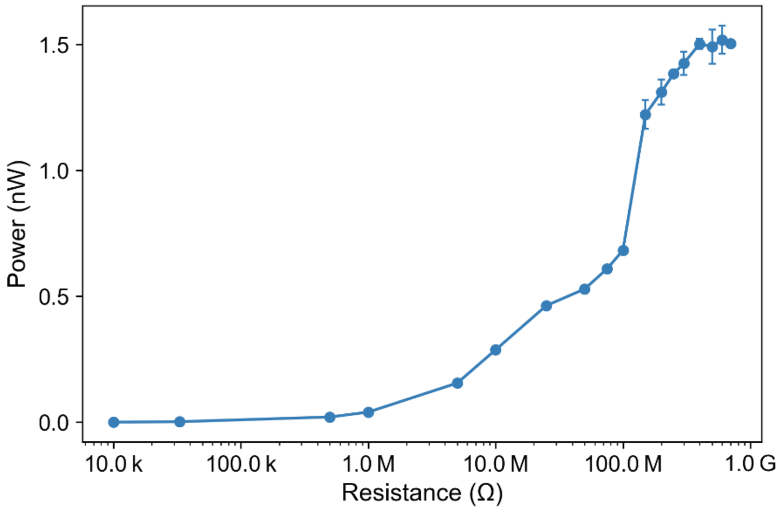

Figure S9. RMS power output of CNT yarn without PVDF fibers coating.

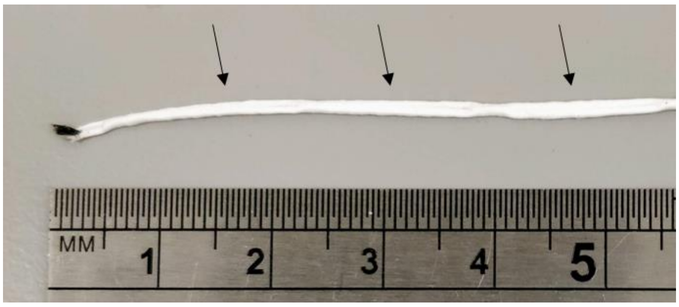

Figure S10. Photograph of triboelectric yarn after 200,000 tapping cycles. The arrows indicate the areas where tapping occurred. Note the increase in yarn diameter due to the deformation of the PVDF fibers.

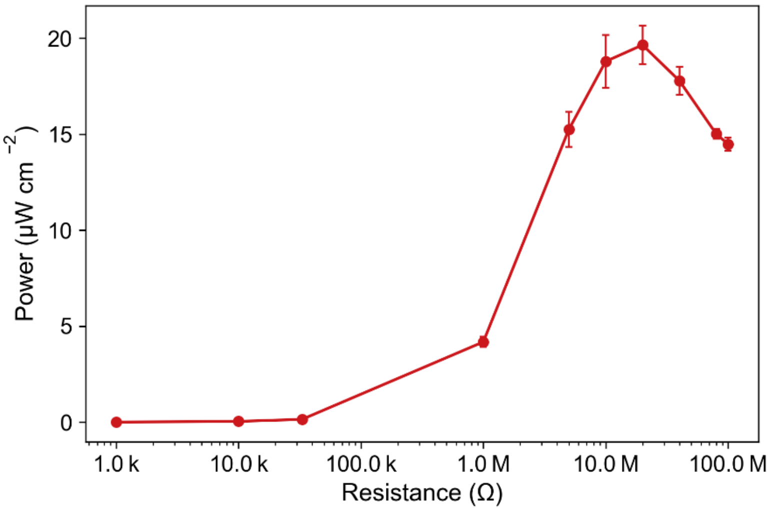

Figure S11. Peak power density of triboelectric yarn after 200,000 fatigue cycles.

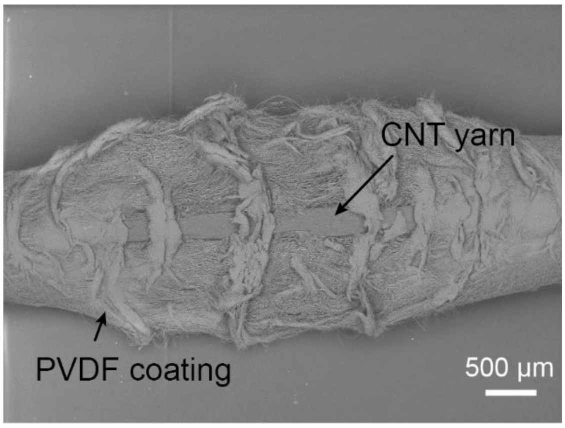

Figure S12. SEM image of the triboelectric yarn after the wear resistance test.

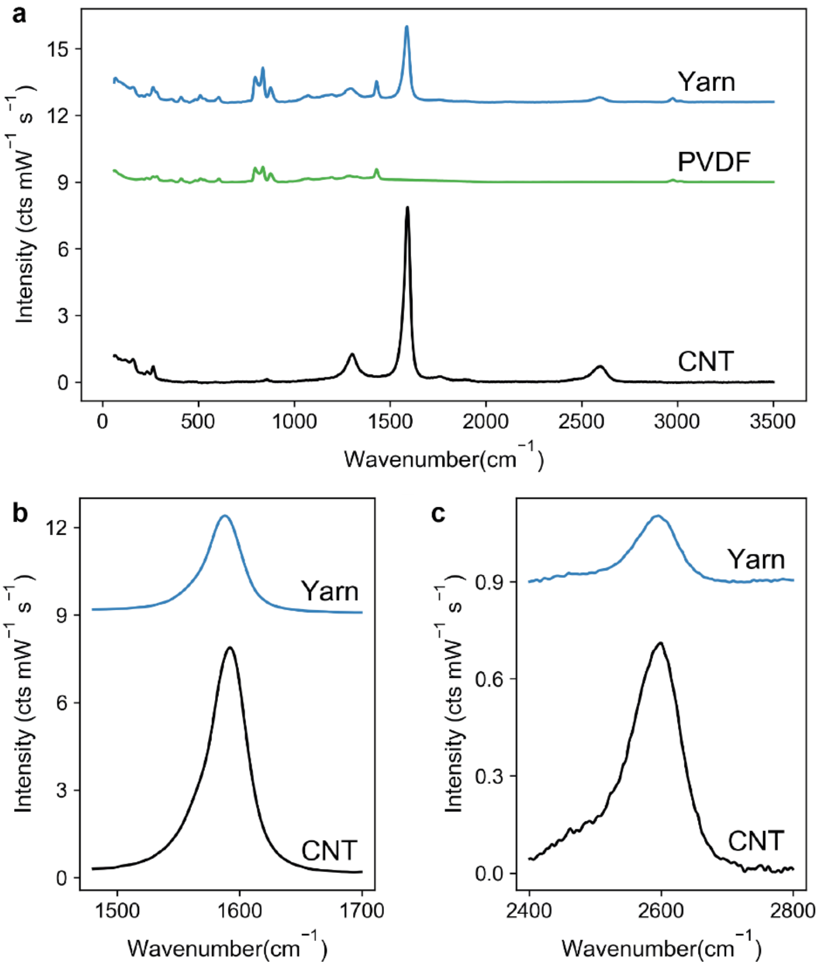

Figure S13. Raman spectra of CNT yarn, PVDF fibers and the triboelectric yarn. a) Spectra of CNT yarn, PVDF fibers and the triboelectric yarn. Spectra are the mean of three spectra within the same material. All spectra are normalized, offset and background subtracted for visual

clarity. b) Spectra of G peak of CNT yarn and triboelectric yarn. c) Spectra of 2D peak of CNT yarn and triboelectric yarn.

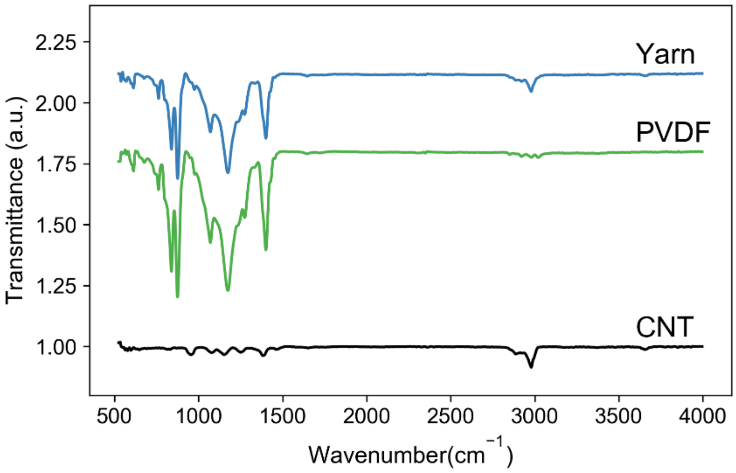

Figure S14. FTIR spectra of CNT yarn, PVDF fibers and the triboelectric yarn. a) Spectra of CNT yarn, PVDF fibers and the triboelectric yarn. All spectra have been normalized, atmospheric corrected, offset and background subtracted for visual clarity.

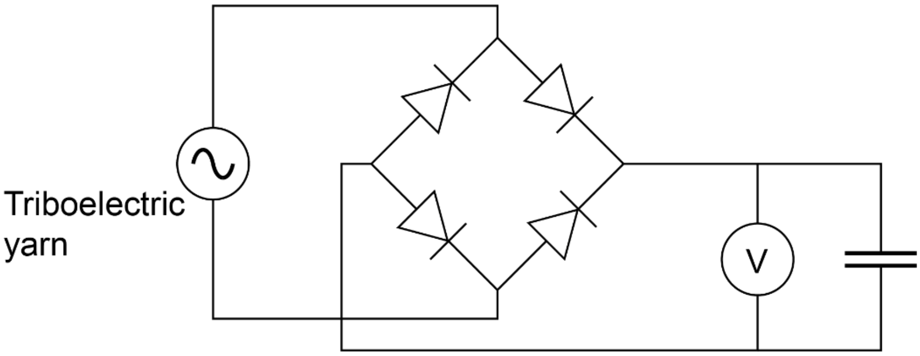

Figure S15. A schematic of the rectifying circuit used to charge the capacitors using the triboelectric yarn.

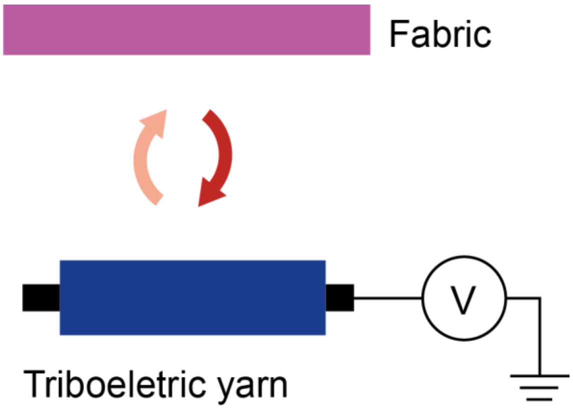

Figure S16. A schematic of the circuit used to detect arm motion and finger bending.

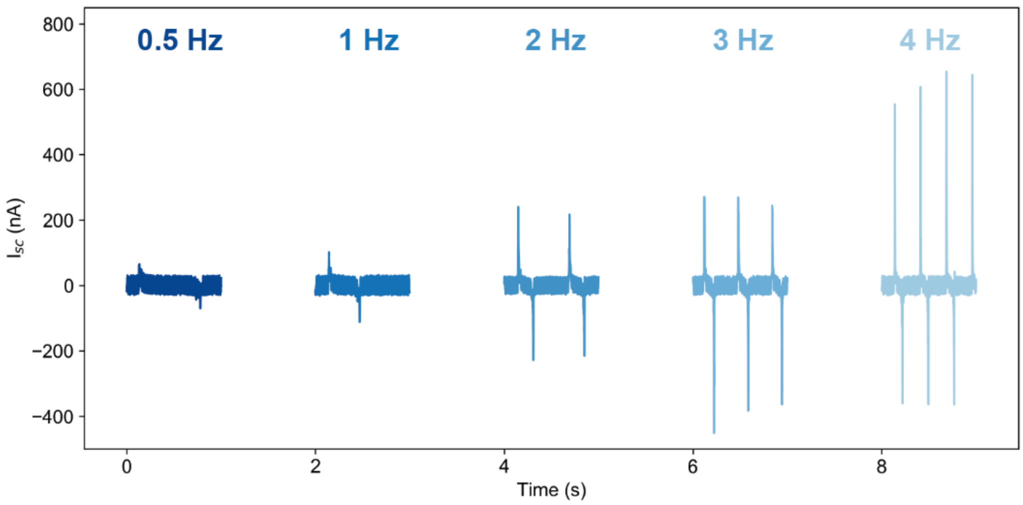

Figure S17. I_{sc} of the triboelectric yarn with increasing motion frequency.
